# Open stent graft repair with upper-half Sternotomy for blunt thoracic aortic injury: a case report

**DOI:** 10.1186/s13019-017-0667-4

**Published:** 2017-11-29

**Authors:** Toshinori Komatsu, Tamaki Takano, Hiromu Kehara, Megumi Fuke, Takamitsu Terasaki, Masayuki Sakaguchi

**Affiliations:** Department of Cardiovascular Surgery, Nagano Red Cross Hospital, 5-22-1 Wakasato, Nagano City, Nagano, 380-8582 Japan

**Keywords:** Open stent graft, Blunt thoracic aortic injury, Aortic dissection, Thoracic endovascular aortic repair, Multiple trauma

## Abstract

**Background:**

Thoracic endovascular aortic repair is now widely applied to the treatment of blunt aortic injury. However, its long-term outcomes remain unclear. Endoleakage and migration might occur in the long term, especially when younger patients undergo endovascular aortic repair. In open stent grafting, the proximal end of the open stent graft is directly sutured to the native aorta, which may reduce the risk of endoleakage and migration. We applied open stent grafting to the treatment of blunt aortic injury in the subacute phase and herein report the patient’s clinical course.

**Case Presentation:**

A 20-year-old man with a developmental disorder collided with a steel tower while skiing. He was transferred to our hospital by helicopter. X-ray examination and computed tomography revealed fractures of left humeral head and femoral neck and aortic isthmus dissection. We did not perform an acute-phase operation because of the presence of multiple trauma and instead performed open stent grafting with an upper-half sternotomy 42 days after the injury. He recovered uneventfully without psychological problems other than his preexisting developmental disorder. No endoleakage or aneurysm was observed during an 18-month follow-up period.

**Conclusions:**

Open stent grafting might be an alternative to open surgery and thoracic endovascular aortic repair for blunt chest trauma, although intensive follow-up is needed.

## Background

Blunt thoracic aortic injuries (BTAIs) occur at the aortic isthmus just distal to the left subclavian artery in 80%–96% of patients [1, 2]. The primary mechanism associated with BTAI is sudden deceleration, often associated with automobile or plane crashes, falls, or vehicle vs. pedestrian accidents [3]. Eighty percent of patients with BTAI will die before reaching a hospital [4], and BTAI is thus considered a surgical emergency. However, it is sometimes difficult to determine the treatment priority because BTAI is often complicated by other serious injuries. BTAI can be repaired by open surgery or thoracic endovascular aortic repair (TEVAR). TEVAR is now widely applied to the treatment of BTAI, but its long-term outcomes remain unclear. Open stent grafting (OSG) may have a lower risk of endoleakage and migration than TEVAR because the proximal end of the graft is sutured to the native aorta. However, the long-term outcomes of this procedure are also unknown. We herein report a case of BTAI treated with OSG in the subacute phase.

## Case Presentation

**Fig. 1:**
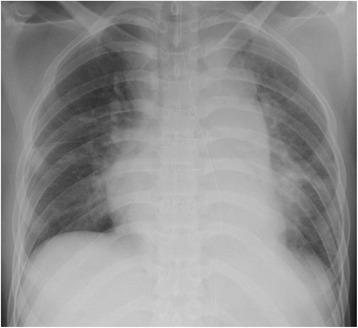
Chest X-ray showed a wide mediastinum, enlarged aortic knob, rightward deviation of the nasogastric tube, and a smudgy aortopulmonary window

**Fig. 2:**
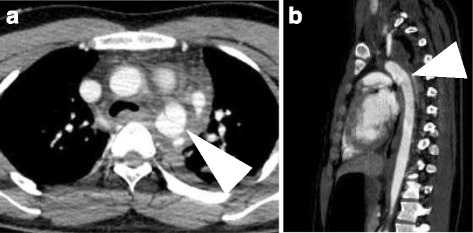
Preoperative contrast-enhanced computed tomography. **a** Horizontal dislocation. **b** Sagittal section. The arrowhead shows dissection in zones 3 to 4 of the thoracic aorta

**Fig. 3:**
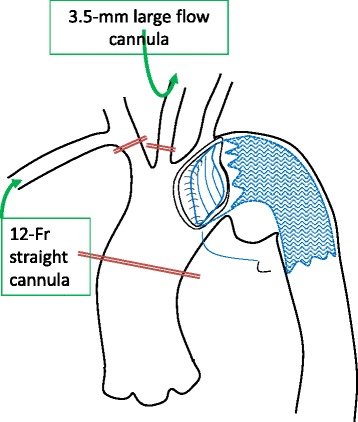
Schematic drawing of our open stent procedure

**Fig. 4:**
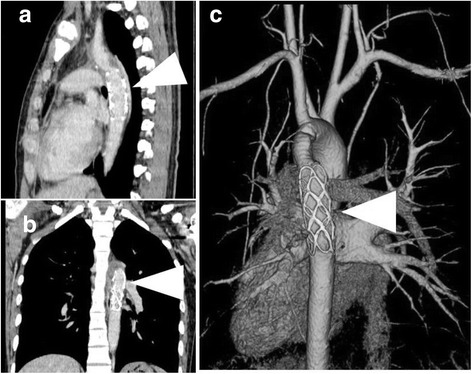
Postoperative contrast-enhanced computed tomography. **a** Horizontal dislocation. **b** Sagittal section. **c** Three-dimensional computed tomography. The arrowhead shows the stent graft

**Fig. 5:**
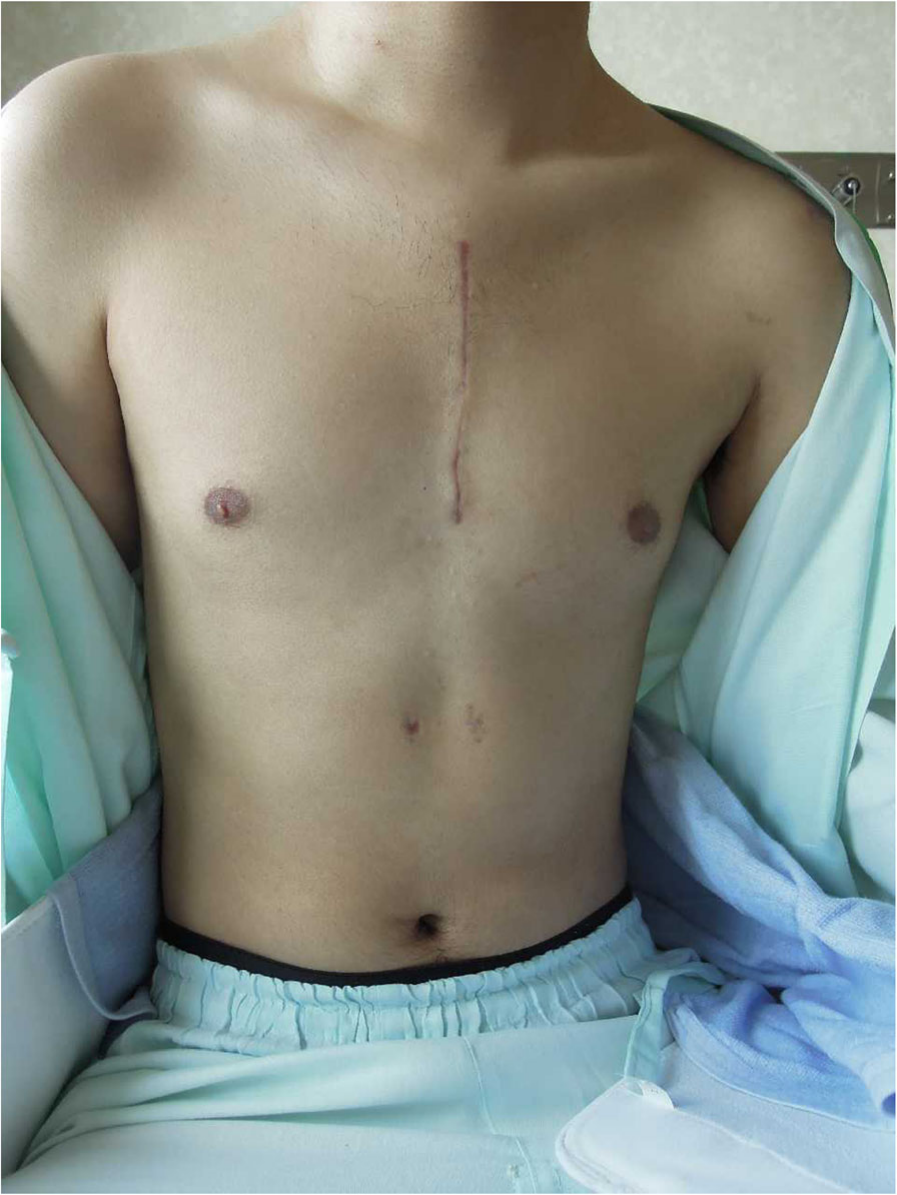
Surgical wound 43 days after the open stent grafting with upper-half sternotomy

A 20-year-old man with a developmental disorder collided with a steel tower while downhill skiing. The patient sustained a whole-body contusion and was airlifted to our hospital. Upon arrival, his blood pressure was 99/65 mmHg and heart rate was 87 bpm. His consciousness was lucid, and he complained of pain in the left upper arm and right thigh. Physical examination revealed subcutaneous emphysema and tenderness in the left anterior chest, although he did not complain of dyspnea. A chest X-ray showed a wide mediastinum, enlarged aortic knob, rightward deviation of the nasogastric tube, and a smudgy aortopulmonary window, which led us to suspect thoracic aorta injury (Fig. 1). Contrast-enhanced computed tomography confirmed dissection in zone 3 to 4 of the thoracic aorta, a hematoma in the left supraclavicular fossa and mediastinum, and the presence of hemothorax (Fig. 2a, b) without active bleeding. X-ray examination of the limbs and pelvis revealed fractures of left humeral head and femoral neck. Blood examination showed a slightly elevated white blood cell count and slightly elevated aspartate aminotransferase, lactate dehydrogenase, and creatine phosphokinase concentrations. The D-dimer concentration was 15.3 μg/mL. We chose conservative therapy for the acute aortic dissection because the patient was hemodynamically stable and the multiple fractures might have caused uncontrollable bleeding during heparinization for cardiopulmonary bypass. His blood pressure was strictly controlled at <120 mmHg by continuous intravenous administration of nicardipine and nitroglycerin. Eight days after admission, the fractures of the left humeral head and femoral neck were surgically repaired. We then surgically repaired the aortic dissection 42 days after admission. An upper-half sternotomy was performed until the fourth intercostal space was reached, and cardiopulmonary bypass was initiated with a 29-Fr right atrial cannula, a 10-Fr common carotid arterial cannula, and a 12-Fr axillary arterial cannula. After induction of moderate hypothermia at a rectal temperature of 28 °C, selective antegrade cerebral perfusion was begun. An open stent graft (J-Graft; Japan Lifeline, Tokyo, Japan) with a 21-mm diameter and 6-cm length was inserted via an aortotomy on the aortic arch just distal to the left subclavian artery during circulatory arrest. The proximal end of the stent was sutured to the aortic wall with 4–0 polypropylene by continuous suture (Fig. 3). Then, the aortotomy was closed by continuous suture. The total operation time was 345 min, the cardiopulmonary bypass time was 192 min, and the arrest time was 53 min. The patient made steady progress postoperatively; he was weaned from the ventilator 3 h after the surgery. He began orthopedic rehabilitation on postoperative day 19. Computed tomography revealed no endovascular stenosis or endoleaks (Fig. 4). He was discharged from the hospital on postoperative day 43 (Fig. 5).

## Discussion

BTAI had a reported incidence of 0.3% in a trauma registry study that reviewed 5838 pedestrians injured by automobiles [5]. Another study found an overall incidence of BTAI of 6.8 per 10,000 occupants of motor vehicles involved in crashes, reported in the National Automotive Sampling System in the United States [6]. One third of patients with fatal blunt trauma have thoracic aortic injuries, though the majority of deaths occur at the scene of the injury. An autopsy study revealed that 34% of victims who received blunt trauma had a thoracic aortic injury [7]. BTAI is the second-leading cause of death in trauma patients, and 80% of patients with BTAI will die before reaching a trauma center [4]. Among patients who survive to arrive at hospital, 50% will die within 24 h. This significant mortality rate is related to the high incidence (40%) of severe associated injuries [4].

The Society for Vascular Surgery classified BTAI on the basis of severity as follows: grade I, intimal tear; grade II, intramural hematoma; grade III, aortic pseudoaneurysm; and grade IV, free rupture. The Society’s guidelines suggest urgent TEVAR for grade II–IV BTAI, and expectant management with serial imaging for grade I injuries [8]. TEVAR has recently shown good outcomes in patients with significant comorbidities and concomitant injuries [8, 9] However, no grafts with an appropriate caliber are usually available for otherwise normal aortas in the trauma setting [10]. TEVAR cannot secure enough landing zones in some cases. The long-term effects of endovascular stenting require further assessment in the predominantly younger population of patients who sustain BTAI. In the present case, we chose OSG to fix the stent more securely than possible with TEVAR because the patient was 20 years old and careful long-term follow-up was needed. OSG, which has been described as the frozen elephant trunk technique in Europe and the United States, has been especially useful for aortic arch aneurysm repair in recent years. The following three advantages of OSG are particularly noteworthy: the need for a distal anastomotic suture is eliminated, the need for a left thoracotomy is eliminated, and detachment is minimized [11]. Additionally, in contrast to open surgery, the discharge period after OSG is early and rehabilitation can be started immediately. In the present case, the patient was extubated 3 h postoperatively, and no coagulopathy was observed during or after the operation. He was discharged from the hospital without psychological problems other than his preexisting developmental disorder. However, special care was taken during the postoperative follow-up period in this case because no previous cases of OSG repair in the treatment of BTAI have been reported.

We performed OSG 42 days after the onset of BTAI because the patient had multiple trauma. Blood pressure control was important to reduce the risk of rupture after the aortic dissection during the conservative therapy. Postoperative outcomes appear to be better for patients who undergo delayed surgical repair than for those who undergo immediate repair [1]. The Society for Vascular Surgery published clinical practice guidelines stating that TEVAR should be preferentially used over open repair and nonoperative management and that repair should be urgently performed within 24 h after the injury [12]. However, Di Eusanio et al. [13] and Challoumas and Dimitrakakis [14] stated that delayed repair 15 to 302 days after the injury was associated with satisfactory outcomes. The timing of repair depends on the extent of injury of the thoracic aorta and other organs. We found no reports showing a definitive relationship between the degree of trauma and the timing of repair. Further studies are mandatory to determine the appropriate timing of repair in patients with BTAI, although the timing of surgery was appropriate in the present case.

## Conclusions

OSG with upper-half sternotomy was performed for treatment of BTAI, and the postoperative course was uneventful. OSG might be an alternative to open surgery and TEVAR in patients with BTAI, although intensive follow-up should be performed.

## Abbreviations

BTAIs: Blunt thoracic aortic injuries
OSG: Open stent grafting
TEVAR: Thoracic endovascular aortic repair
